# Senescence Marker Protein-30 (SMP30) Deficiency Impairs Myocardium-Induced Dilation of Coronary Arterioles Associated with Reactive Oxygen Species

**DOI:** 10.3390/ijms14059408

**Published:** 2013-04-29

**Authors:** Hiroyuki Mizukami, Shu-ichi Saitoh, Hirofumi Machii, Shinya Yamada, Yasuto Hoshino, Tomofumi Misaka, Akihito Ishigami, Yasuchika Takeishi

**Affiliations:** 1 Department of Cardiology and Hematology, Fukushima Medical University, 1 Hikarigaoka, Fukushima 960-1295, Japan; E-Mails: hirohiro@fmu.ac.jp (H.Mi.); machii@fmu.ac.jp (H.Ma.); smyyamada0124@yahoo.co.jp (S.Y.); capture6285@yahoo.co.jp (Y.H.); misaka83@fmu.ac.jp (T.M.); takeishi@fmu.ac.jp (Y.T.); 2 Molecular Regulation of Aging, Tokyo Metropolitan Institute of Gerontology, 35-2 Sakae-cho, Itabashi-ku, Tokyo, 173-0015, Japan; E-Mail: ishigami@tmig.or.jp

**Keywords:** coronary circulation, oxidant stress, SMP30, cardiac myocyte, coronary arterioles, vasomotor tone

## Abstract

Senescence marker protein-30 (SMP30) decreases with aging. Mice with SMP30 deficiency, a model of aging, have a short lifespan with increased oxidant stress. To elucidate SMP30’s effect on coronary circulation derived from myocytes, we measured the changes in the diameter of isolated coronary arterioles in wild-type (WT) mice exposed to supernatant collected from isolated paced cardiac myocytes from SMP30 KO or WT mice. Pacing increased hydrogen peroxide in myocytes, and hydrogen peroxide was greater in SMP30 KO myocytes compared to WT myocytes. Antimycin enhanced and FCCP (oxidative phosphorylation uncoupler in mitochondria) decreased superoxide production in both groups. Addition of supernatant from stimulated myocytes, either SMP30 KO or WT, caused vasodilation. The degree of the vasodilation response to supernatant was smaller in SMP30 KO mice compared to WT mice. Administration of catalase to arterioles eliminated vasodilation in myocyte supernatant of WT mice and converted vasodilation to vasoconstriction in myocyte supernatant of SMP30 KO mice. This vasoconstriction was eliminated by olmesartan, an angiotensin II receptor antagonist. Thus, SMP30 deficiency combined with oxidant stress increases angiotensin and hydrogen peroxide release from cardiac myocytes. SMP30 plays an important role in the regulation of coronary vascular tone by myocardium.

## 1. Introduction

**Figure A1:**
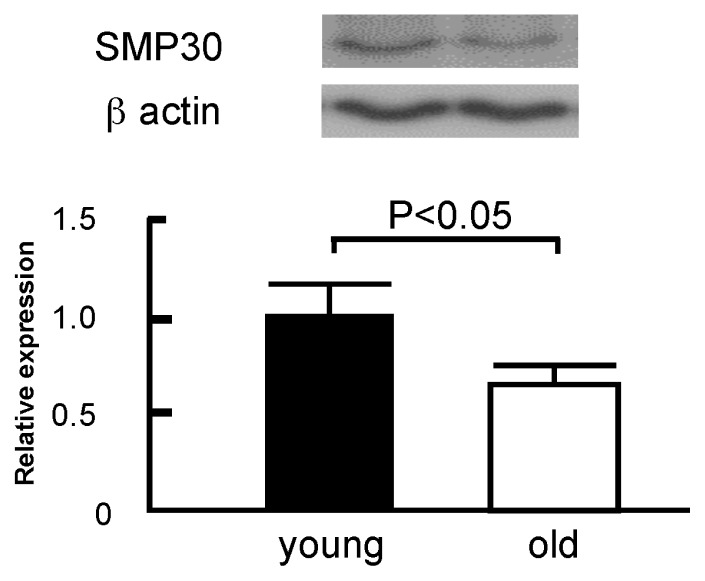
SMP30 expression in myocardium of young and old mice. We measured SMP30 expression in myocardium of young (3 months old) and old (12 months old) mice (*n* = 5 each) by western blotting. Total protein was extracted from the snap-frozen left ventricle using Cell Lysis Buffer (Cell Signaling Technology, Inc., Beverly, MA, USA) with Protease Inhibitor Cocktail (BD Biosciences, San Jose, CA, USA) as previously reported [33]. Protein concentration was determined by protein assay (DC Protein Assay Kit, Bio-Rad Laboratories, Inc., Hercules, CA, USA). Equal amounts (20 μg) of the protein samples were separated by sodium dodecyl sulfate-polyacrylamide gel electrophoresis (SDS-PAGE, 5%–20%) and transferred onto polyvinylidene difluoride membranes (ATTO Co., Tokyo, Japan). The primary antibodies were anti-SMP30 (SHIMA Laboratories Co. Ltd., Tokyo, Japan) and mouse anti-β-actin (Santa Cruz Biotechnology Inc., California, CA, USA). The secondary antibodies were goat anti-rabbit IgG-horseradish peroxidase and goat anti-mouse IgG-horseradish peroxidase (Santa Cruz Biotechnology Inc., California, CA, USA). The signals from immunoreactive bands were visualized by an Amersham ECL system (Amersham Pharmacia Biotech UK Ltd., Buckinghamshire, UK) and quantified using densitometric analysis. SMP30 expression was lower in myocardium of old mice (12 months old) than young mice (3 months old). Data are expressed as the mean ± S.D.

Senescence marker protein-30 (SMP30), a 34-kDa protein, is a novel molecule whose expression decreases with age in a sex-independent manner. SMP30 protects cellular functions from age-associated deterioration [1] (Appendix Figure A1). In humans, the SMP30 gene is located in the p11.3–q11.2 segment of the X chromosome [2]. In mice, SMP30 transcripts are detected in various organs, including the liver, kidney, cerebrum, testis, and lung. SMP30 knock-out (KO) mice have a short lifespan and lack ascorbic acid biosynthesis [1,3]. Therefore, we speculated that the depletion of SMP30 closely relates to the increase of oxidant stress that occurs with aging. Several studies have investigated the relationship between aging and oxidant stress and the cytoprotective role of SMP30 against oxidant stress in cell lines [1,4]. On the other hand, the increase of oxidant stress with aging plays an important role in the development of coronary artery disease, during which vascular resistance increases, leading to impairments in coronary blood flow and flow reserve [5,6]. Coronary vasomotor tone is regulated through unique mechanisms, such as endothelium-dependent modulation, myogenic regulation and neural control [7]. Above all, the principal driving force for the control of coronary blood flow is myocardial metabolism [8]. However, the underlying mechanism that drives the aging-related, redox-stress-related impairment of myocardium-induced coronary flow regulation is unclear.

We have reported that superoxide is produced by cardiac myocytes in proportion to cardiac metabolism, which leads to the production of vasoactive hydrogen peroxide (H_2_O_2_) [9]. Moreover, in mimicking a heart failure episode, cardiac myocytes release angiotensin, which leads to vasoconstriction [10]. We hypothesized that oxidant stress related to downregulation of SMP30 would be a trigger in the modification of cardiac myocyte function; *i.e.*, oxidant stress can alter the generation of cardiac myocyte metabolites. However, in the whole heart, it is difficult to separate specific metabolic regulators of coronary artery tone from other regulatory systems, such as endothelium-dependent regulation, autonomic nerve-dependent regulation, and flow-dependent regulation. We have therefore adopted a procedure using isolated cardiac myocytes and single arterioles to evaluate the specific effect of metabolic regulation on the coronary blood-flow system [9–11]. Using this system, we investigated SMP30’s effect on coronary vasoactive responses to cardiac myocyte metabolites and examined the effect of myocardium-dependent regulation of coronary vascular tone in response to oxidant stress in SMP30 KO mice. We measured the generation of H_2_O_2_ and superoxide in cardiac myocytes of SMP30 KO mice and the change in coronary arterial tone induced by myocyte metabolites.

## 2. Results

### 2.1. Cardiac Myocyte Cell Viability

To examine cell viability in isolated myocytes, we performed MTT assays. Isolated cardiac myocyte cell viability did not change until after 20 min of electrical stimulation in wild-type (WT) and SMP30 KO mice (WT mice: non-stimulated, 0.48 ± 0.06 O.D.; 20 min stimulation, 0.46 ± 0.05 O.D.; SMP30 KO mice: non-stimulated, 0.46 ± 0.08 O.D.; 20 min stimulation, 0.45 ± 0.06 O.D.; *n* = 12 each).

Viability of the cardiac myocytes was also determined by trypan blue exclusion and rod-shaped configuration in directly. On average, >85% of the cells exhibited a rod-like configuration.

### 2.2. Generation of O_2_^−^ and H_2_O_2_ and NADPH Oxidase Activity in Cardiac Myocytes

**Figure 1:**
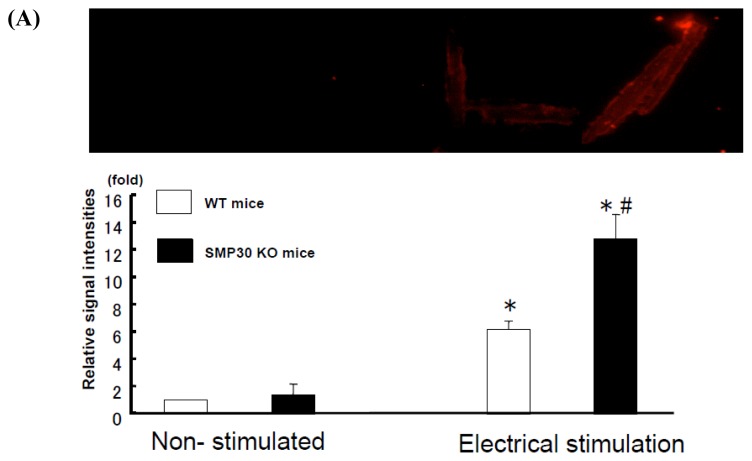

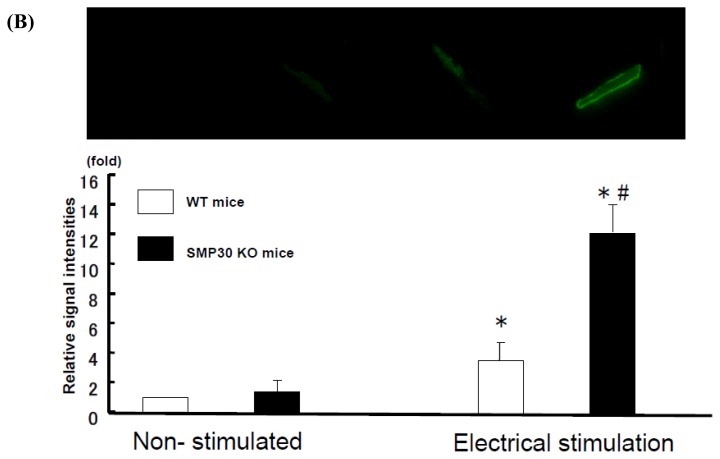
DHE and DCF staining in cardiac myocytes. Representative DHE (**A**) and DCF (**B**) staining in cardiac myocytes (Upper panel). Summary data of DHE and DCF staining in cardiac myocytes (Lower panel). The signals of DHE and DCF increased with electrical stimulation. DHE and DCF signals were more potent in SMP30 KO cardiac myocytes compared to WT cardiac myocytes with electrical stimulation. Values are expressed as the mean ± S.E.M. * *p* < 0.01 *vs.* non-stimulated, # *p* < 0.01 *vs.* WT mice in same staining (*n* = 12 each).

To examine the generation of O_2_^−^ or H_2_O_2_, we measured the signal intensity of dihydroethidium (DHE)- or dichlorodihydro-fluorescein (DCF)-stained isolated cardiac myocytes. The signals of DHE and DCF staining were enhanced with the increase of electrical stimulation in cardiac myocytes (DHE: WT mice, 6.2 ± 0.6-fold; SMP30 KO mice, 12.8 ± 1.8-fold; DCF: WT mice, 3.5 ± 1.2-fold; SMP30 KO mice, 12.2 ± 1.8-fold; *n* = 12 each) compared to non-stimulation for 20 min (*p* < 0.01 for each) (Figure 1A,B).

**Figure 2:**
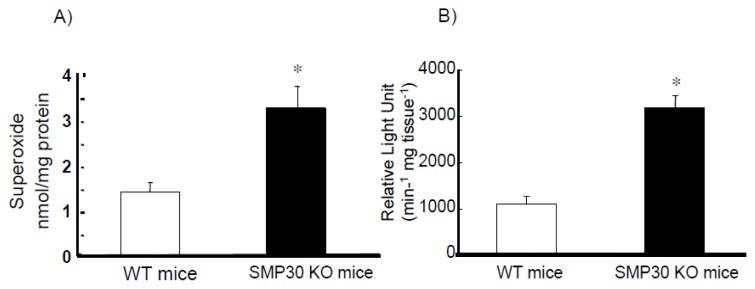
Effect of SMP30 deficiency on generation of superoxide and activity of NADPH oxidase in cardiac myocytes under electrical stimulation. Generation of superoxide (**A**) was measured by HPLC. NADPH oxidase activity was measured by lucigenin luminescence; (**B**) The levels of superoxide and NADPH oxidase activity were greater in SMP30 KO mice compared to WT mice under electrical stimulation. Values were expressed as the mean ± S.E.M. * *p* < 0.01 *vs.* without agents. *n* = 8, each.

Superoxide in cardiac myocytes was also measured by HPLC. More superoxide was generated in SMP30 KO cardiomyocytes compared to WT under electrical stimulation (Figure 2A). Further, NADPH oxidase activity was greater in SMP30 KO cardiomyocytes compared to WT under electrical stimulation (Figure 2B).

**Figure 3:**
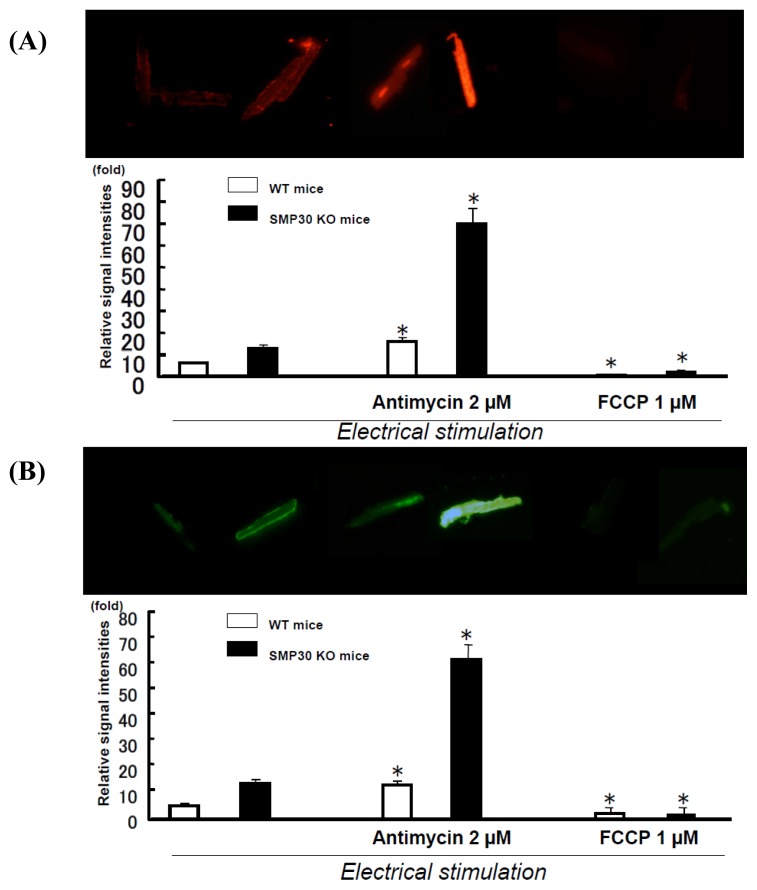
DHE and DCF staining in paced cardiac myocytes with antimycin or FCCP. Representative DHE (**A**) and DCF staining (**B**) in cardiac myocytes (Upper panel). Summary data of DHE and DCF staining in cardiac myocytes (Lower panel). The signals of DHE and DCF were enhanced by antimycin (2 μM) and attenuated by FCCP (1 μM). Values are expressed as the mean ± S.E.M. * *p* < 0.01 *vs.* without agents (*n* = 12 each).

In stimulated myocytes, antimycin significantly increased the signals of DHE and DCF. In contrast, *p*-trifluoromethoxy carbonyl cyanide phenyl hydrazone (FCCP) decreased these signals (Figure 3A,B).

**Figure 4:**
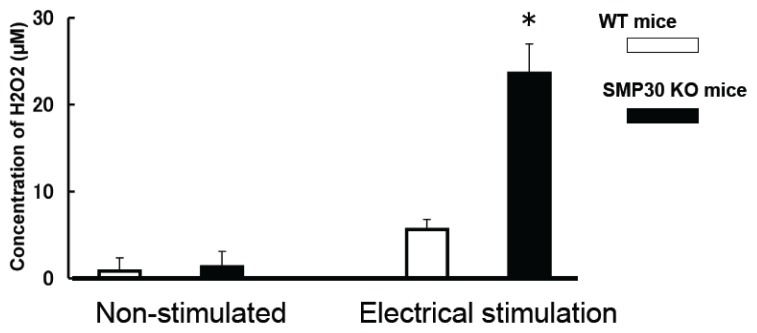
The level of H_2_O_2_ in cardiac myocyte supernatant. The concentration of H_2_O_2_ in the cardiac myocyte supernatant increased with pacing. H_2_O_2_ in SMP30 KO myocytes was higher than in WT cardiac myocytes. Values are expressed as the mean ± S.E.M. * *p* < 0.01 *vs.* WT mice (*n* = 12 each).

The concentration of H_2_O_2_ in both WT and SMP30 KO myocyte supernatants increased with the electrical stimulation. However, the H_2_O_2_ level after 20 min stimulation in SMP30 KO myocyte supernatant was higher than that of WT myocyte supernatant (23.8 ± 3.4 *vs.* 5.6 ± 1.2 μM, *p* < 0.01) (Figure 4). We also measured H_2_O_2_ in a stimulated buffer without myocytes, but the concentration was too low for detection by our system.

### 2.3. Vitamin C Level and Catalase Activity

**Figure 5:**
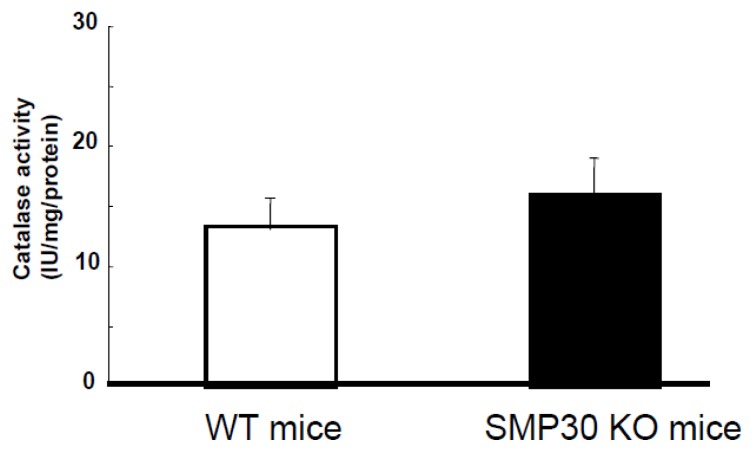
Catalase activity. Catalase activity was not different between WT and SMP30 KO myocardium. Values are expressed as the mean ± S.E.M (*n* = 12 each).

Mice were given food including vitamin C because SMP30-deficient mice cannot synthesize vitamin C *in vivo.* Vitamin C levels in the left ventricle did not differ between SMP30 KO and WT mice (0.11 ± 0.05 μmol/g tissue *vs.* 0.13 ± 0.06 μmol/g tissue, *n* = 10 each). Catalase activity in myocardium was not different between SMP30 KO and WT mice (Figure 5).

### 2.4. Superoxide Anion Radical (O_2_^−^) Scavenging Activity (SOD Activity)

**Figure 6:**
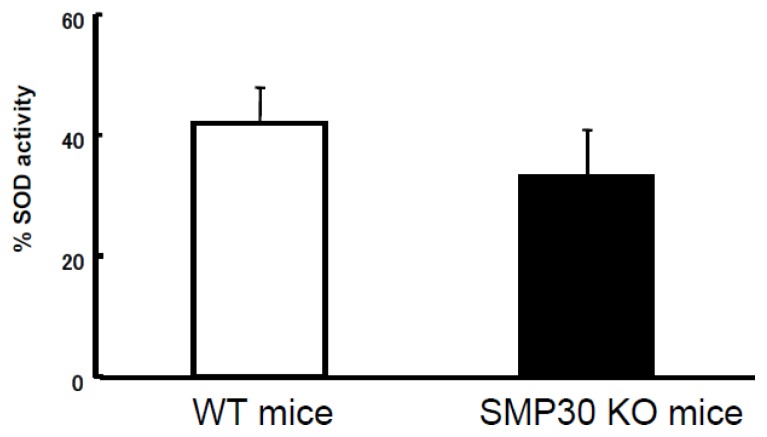
SOD activity. SOD activity was not different between WT and SMP30 KO myocardia. Values are expressed as the mean ± S.E.M (*n* = 12 each).

SOD activity was not different between cardiac myocytes isolated from SMP30 KO and WT mice (Figure 6).

### 2.5. Vasodilative and Vasoconstrictive Properties in Supernatant of Stimulated Myocytes

**Figure 7:**
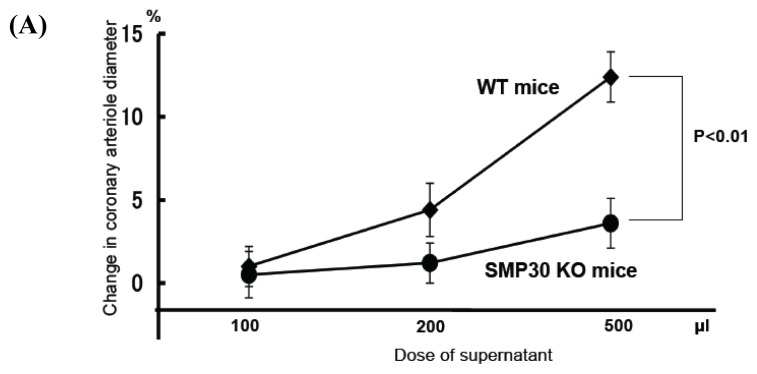

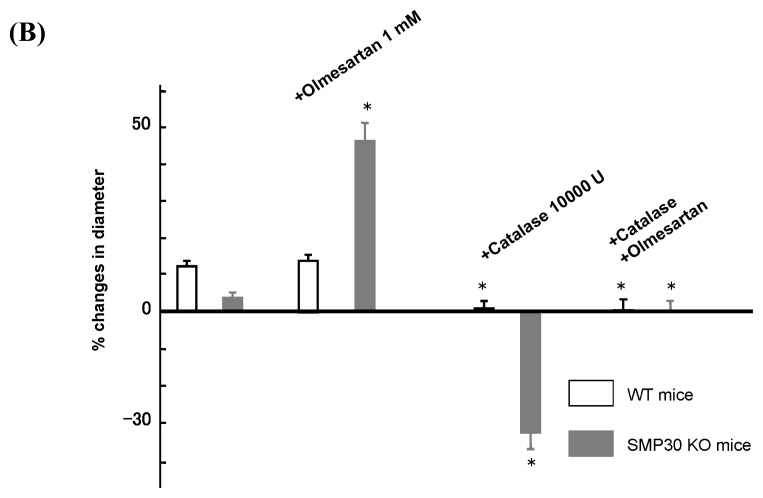
Vasodilation induced by the supernatant from stimulated cardiac myocytes. The supernatant of electrically stimulated cardiac myocytes dilated WT coronary arterioles dose-dependently. Vasodilation with WT cardiac myocyte supernatant was more potent compared to that with SMP30 KO cardiac myocyte supernatant (**A**) Administration of olmesartan to coronary arterioles enhanced vasodilation in the SMP30 KO cardiac myocyte supernatant treatment group (500 μL of supernatant). Administration of catalase to coronary arterioles converted vasodilation to vasoconstriction in the SMP30 KO cardiac myocyte supernatant treatment group and eliminated vasodilation in the WT cardiac myocyte supernatant treatment group. Treatment with olmesartan in addition to catalase in the vessel bath eliminated vasoconstriction in the SMP 30 KO cardiac myocyte supernatant treatment group; (**B**) Values are expressed as the mean ± S.E.M. *n* = 12 each, * *p* < 0.01 *vs.* without agent.

To elucidate SMP30’s effect on coronary circulation derived from myocytes, we measured the changes in the diameter of isolated coronary arterioles from WT mice in response to supernatant collected from isolated electrically stimulated cardiac myocytes from SMP30 KO or WT mice. Direct administration of 10,000 U catalase or 1 mM olmesartan to the vessel or vessel bath, respectively, did not change the vascular tone (data not shown). Without electrical stimulation, supernatant from the myocytes failed to produce vasodilation, but during an electrical stimulation of 600 bpm, dose-dependent vasodilation was observed in the supernatant. Vasodilation with WT myocyte supernatant was more potent than with SMP30 KO myocyte supernatant in coronary arterioles (response to 500 μL supernatant of cardiac myocytes: WT mice, 12.4% ± 1.5%; SMP30 KO mice, 3.6% ± 1.5%; *n* = 12; *p* < 0.01) (Figure 7A). Administration of catalase to arterioles converted vasodilation to vasoconstriction in the SMP30 KO cardiac myocyte supernatant treatment group (response to 500 μL supernatant: –32.8% ± 4.5%, *n* = 12, *p* < 0.01 *vs.* without catalase), and this vasoconstriction was eliminated by additional treatment with olmesartan. In the WT cardiac myocyte supernatant treatment group, administration of catalase eliminated vasodilation (response to 500 μL supernatant: 1.0% ± 1.2%, *n* = 12, *p* < 0.01 *vs.* without catalase) (Figure 7B).

## 3. Discussion

This is the first study to demonstrate that SMP30 deficiency alters the metabolic properties of cardiac myocytes, which alters the coronary vascular tone. Deficiency of SMP30 enhanced activity of NADPH oxidase in cardiac myocytes, as reported by Son *et al.* that SMP30 deficiency causes oxidant stress through NADPH oxidase in brain [12]. In addition, lack of SMP30 increases the production of H_2_O_2_ and angiotensin in cardiac myocytes, effects that are related to generation of reactive oxygen species. Then, we concluded that mitochondrial electron transportation system plays an important role to produce H_2_O_2_, which is a metabolic vasodilator, as well as NADPH oxidase pathway in cardiac myocytes. This conclusion is strengthened by the results of studies using antimycin and FCCP. Antimycin, an inhibitor of mitochondrial electron transportation complex III, increases the generation of superoxide [13], which then is converted to H_2_O_2_. FCCP is an ionophore that decreases the mitochondrial protonmotive force, which then uncouples mitochondrial electron transport and reduces superoxide production. Our observations also show that the increase in superoxide during pacing of myocytes was decreased by FCCP, further supporting the concept that increased electron transport is the basis for increased superoxide production and thus H_2_O_2_ production during heightened myocardial metabolism.

Mitochondrial oxidant production increases with age [14]. Using DHE fluorescence and HPLC studies, we observed that superoxide production increased in SMP30 KO cardiac myocytes compared to WT cardiac myocytes. In addition, catalase and SOD activity did not change in either group’s cardiac myocytes, a finding that was similarly reported by Son *et al.* [12]. From these results, it could be speculated that SMP30 regulates the mitochondrial electron transport system and its deficiency augments superoxide generation through electron flow in mitochondria.

As SMP30 deficiency increases reactive oxygen species in mitochondria, calcium may be a key regulator of this process. SMP30 is associated with intracellular calcium homeostasis, regulating [Ca^2+^]_i_ by modulating plasma membrane Ca^2+^-pumping activity, and SMP30 has cytoprotective roles against intracellular calcium elevation in kidney, liver, and brain [12,15]. Overexpression of SMP30 increases Ca^2+^ storage capacity by increasing SERCA level and decreasing the amount of Ca^2+^ released into the cytoplasm. In contrast, during the aging process, SMP30 downregulation reduces SERCA level, as well as directly reducing SERCA activity [16]. Therefore, we speculate that calcium overload in mitochondria is a trigger that increases superoxide production in SMP30 KO mice, as reported previously, though further study is needed [17].

On the other hand, a lack of SMP30 renders cells unable to synthesize the potent antioxidant vitamin C. We fed our mouse model a diet that included vitamin C, and the level of vitamin C in the left ventricle did not differ between WT and SMP30 KO mice. Vitamin C deficiency causes a small increase in the generation of reactive oxygen stress. Humans cannot synthesize vitamin C, so it must be provided in the diet. Thus, SMP30 KO mice may be an acceptable model of human aging and of the downregulation of SMP30 in aging humans.

We have previously reported that rat cardiac myocytes in heart failure, ischemia, and senescence states release angiotensin, which is mediated by oxidant stress [10,18,19]. In this study, a decrease of hydrogen peroxide by catalase treatment augmented the vasoconstriction caused by electrically stimulated myocyte supernatant in SMP30 KO mice. Moreover, vasodilation was converted to vasoconstriction by catalase, an effect that was eliminated by treatment with angiotensin II type I receptor antagonist. These results suggest that electrically stimulated cardiac myocytes generate O_2_^−^, which mediates angiotensin II release and converts hydrogen peroxide. Angiotensin II plays an important role in the deterioration of metabolic coronary flow regulation with aging, providing direct confirmation of the importance of the angiotensin II increase in SMP30 KO myocyte supernatant. We were unable to measure the level of angiotensin production by cardiac myocytes, presumably because the angiotensin concentration was too low to measure. However, several reports show that myocardium releases angiotensin in mice and rats [20–22]. In addition, cardiac senescence is associated with enhanced expression of angiotensin II receptor [23], and age-related increases of angiotensin II have been reported in skeletal muscle in mice [24]. This led us to speculate that even though its concentration was too low to measure, angiotensin, which increases with aging, is sufficient to induce coronary arteriole constriction. Moreover, angiotensin-converting enzyme (ACE) activity may change in cardiac myocytes of SMP 30 KO mice because ACE inhibitors protect cell components from oxidative damage by increasing the enzymatic antioxidant defenses [25]. Thus, studies are needed to clarify the activity of ACE in myocardium of SMP30 KO mice.

## 4. Experimental Section

This investigation conformed to the Guidelines on Animal Experiments of Fukushima Medical University, the Japanese Government Animal Protection and Management Law (No. 105), and the *Guide for the Care and Use of Laboratory Animals* (NIH Publication No. 85–23, revised 1996).

Male SMP30 KO mice were created from C57BL/6 mice by the gene-targeting technique described previously [26]. WT C57BL/6 and SMP30 KO mice (age 8 weeks, body weight, 21.8 ± 2.8 g, heart weight 164 ± 22 mg) were housed and bred in a room at 22 ± 3 °C, with relative humidity 50% ± 10% and a 12-h light-dark cycle. Mice were given food that included vitamin C at 21 mg/100 g (CLEA Japan, Tokyo, Japan) and water *ad libitum*. The mice were anesthetized with sodium pentobarbital (50 mg/kg, ip), and a mid-sternotomy was performed. The hearts were excised and placed in 4 °C buffered physiological salt solution (PSS), then used for microvessel dissection or the isolation of cardiac myocytes.

### 4.1. Isolation of Coronary Arterioles

The bathing solution used for microvessel dissection had the following composition (in mmol/L): 145.0 NaCl, 4.7 KCl, 2.0 CaCl_2_, 1.17 MgSO_4_, 1.2 NaH_2_PO_4_, 5.0 glucose, 2.0 pyruvate, 0.02 EDTA, 3.0 3-(*N*-morpholino)propanesulfonic acid buffer (MOPS), and 1% bovine serum albumin (1 g/100 mL). The solution was buffered to pH 7.4 at 4 °C. A single arteriole was dissected from the left ventricular septum as previously reported [9–11]. A portion of the right ventricle was removed, and coronary arterioles in the left ventricular septum of the appropriate size were located under a dissecting microscope. Each arteriole with its surrounding ventricular muscle was excised, transferred to a temperature-controlled dissection dish (4 °C) containing PSS, and dissected free of the muscle tissue. Side branches were tied off using an 11-0 suture. The vessel was transferred to a Lucite chamber and cannulated at both ends using micropipettes pressurized at 60 mm Hg. The arteriole was tied to each pipette using an 11-0 suture. The PSS used to perfuse the vessels during the experiments was buffered to pH 7.4 at 37 °C. The preparation was then transferred to the stage of an inverted microscope. To assess leaks, the pressure at zero flow was measured, which should equal that in the inflow reservoir pressure when there are no leaks. Any preparations showing leaks were discarded. The vessel was slowly warmed to 37 °C and allowed to develop a spontaneous tone.

### 4.2. Isolation of Cardiac Myocytes

Cardiac myocytes were enzymatically isolated from mouse heart using a modified Piper method [9–11,27]. After excision of the heart, the aorta was cannulated, and the preparation was suspended in a perfusion apparatus. To rinse out residual blood and eliminate contraction, the left ventricle was initially perfused retrogradely from the aorta at 37 °C with oxygenated, calcium-free MOPS buffer (pH 7.40 [titrated with 5 mol/L NaOH]) that contained, in mmol/l: 8 MOPS, 30 taurine, 113 NaCl, 4.7 KCl, 0.6 KH_2_PO_4_, 0.6 Na_2_HPO_4_, 1.2 MgSO_4_, 0.032 Phenol Red, 12 NaHCO_3_, and 10 KHCO_3_. After the cessation of contractile activity, the perfusion was switched to a buffer of the above constituents along with 0.5 mg/mL type 2 collagenase (Worthington, Lakewood, NJ, USA) and 12.5 μmol/L CaCl_2_. After perfusion of the heart for 20 to 25 min and the identification of isolated myocytes in perfusate from the heart, the heart was detached from the perfusion apparatus and placed in a “stop” solution containing the perfusion buffer with 1% BSA and 0.125 mmol/L CaCl_2_. The heart was minced into small pieces that were further titrated in stop buffer. After microscopic confirmation of the presence of myocytes, the cells were filtered and placed in a 10-mL conical tube. CaCl_2_ was added in a series of three steps to arrive at a final concentration of 1.0 mmol/L. Cells were allowed to settle for 20 min, and the supernatant was discarded. Cells were resuspended in the stop buffer with calcium, and small aliquots were then used for cell counts (by a hemocytometer) to enable dilution or concentration (via centrifugation) to a final concentration of 150,000 cells per milliliter.

### 4.3. Electrical Stimulation of Cardiac Myocytes

Suspensions of enzymatically isolated cardiac myocytes were treated in 1.5 mL of MOPS buffer (pH 7.4) under no stimulation or electrical field stimulation at 600 bpm, as described previously [9]. The duration of electrical stimulations was 20 min in a closed chamber. We stimulated the suspension of cardiac myocytes in a handmade, closed, transparent plastic chamber with paired platinum inner nets that faced each other. During electrical stimulation, we confirmed the beating of myocytes with an inverted microscope. When the sample had reached more than 15% damaged myocytes under continuous observation, we excluded it from the following experiment. The survival of the cardiac myocytes was determined with a 3-(4,5-dimethylthiazol-2-yl)-2,5-diphenyltetrazolium bromide (MTT) assay as described in the manufacturer’s protocol (Cat. No. V-13154; Molecular Probes, Eugene, OR). Briefly, the cells were incubated for 3 h in phenol red-free medium containing 0.5% of the yellow mitochondrial dye MTT-positive (MTT+). The amount of blue formazan dye generated from MTT+ was proportional to the number of live cells. The MTT+ reaction was terminated by the addition of DMSO to the medium, followed by incubation for 10 min at 37 °C. The absorbance was read at 540 nm with a spectrophotometer [28]. Moreover, we directly counted cardiac myocytes using the vital dye Trypan Blue. After electrical stimulation for each condition (non-stimulated or stimulated), suspensions were centrifuged (3000*g* for 15 min), and supernatant was immediately snap-frozen in liquid nitrogen. Samples were stored in the freezer until transferred to a vessel, the diameter of which was measured.

### 4.4. DHE and DCF Stainings in Stimulated Cardiac Myocytes

The production of superoxide anion radical (O_2_^−^) and H_2_O_2_ in stimulated cardiac myocytes was measured by staining, respectively, with DHE (10 μM, Sigma Chemical, St. Louis, MO, USA) and DCF (10 μM, Molecular Probes, Eugene, OR, USA). The isolated cardiac myocytes were plated on a slideglass and incubated for 5 min at 37 °C with DHE or DCF. To assess the distribution of DHE or DCF, myocytes were scanned by an Olympus IX71 inverted microscope (Olympus CCD, Tokyo, Japan). In isolated cardiac myocytes, O_2_^−^, and H_2_O_2_ production was then measured in both the non-stimulated myocytes and the 20-min electrically stimulated myocytes with or without antimycin (2 μM) or FCCP (1 μM). We measured the DHE or DCF intensities of 20–24 isolated myocytes in each heart and averaged the data [10]. H_2_O_2_ concentrations in the supernatant of cardiac myocytes in both the non-stimulated and electrically stimulated states were measured by a free radical analyzer (Apollo 4000, WPI Co., Ltd., FL, USA) [9].

### 4.5. Measurements of Superoxide and Activity of NADPH Oxidase in Electrically Stimulated Cardiac Myocytes

Superoxide was measured using an HPLC assay. Isolated cardiac myocytes were washed with Krebs/HEPES buffer; incubated with 50 μM DHE for 30 min at 37 °C and were transferred to methanol for extraction of superoxide-specific product 2-hydroxyethidium (2-OH-E+) and kept at −80°C. Separation of ethidium; 2-OH-E+ and dihydroethidium was performed using a Beckman high-performance liquid chromatography (HPLC) System Gold model with a C-18 reverse-phase column (Nucleosil 250; 4.5 mm; Sigma-Aldrich, St. Louis, MO, USA).

NADPH oxidase activity was quantified by lucigenin-enhanced chemiluminescence [29]. Briefly, NADPH (100 μmol/L) was added to the buffer containing cardiomyocytes (30 μg protein in 500 μL) and lucigenin was injected automatically, at 5 μM to avoid known artifacts when used at higher concentrations. NADPH oxidase activity was calculated by subtracting the basal values from those in the presence of NADPH.

### 4.6. Vitamin C Level and Catalase Activity

Following removal of both atria and the right ventricle, the left ventricle was homogenized in 9 volumes of 100 mM K-phosphate buffer, pH 7.4 [30]. The homogenate was initially centrifuged at 1000*g* for 10 min at 4 °C to remove nuclei and tissue debris. The supernatant was centrifuged again at 30,000*g* for 30 min at 4 °C and stored at −70 °C. Vitamin C in the left ventricle was measured by a high-performance liquid chromatography electrochemical detection method [31]. Myocardial catalase activity was measured according to the Aebi method [32]. The rate of decrease of absorbance at 240 nm upon addition of a known amount of cardiac homogenate was monitored by a spectrophotometer at 25 °C. The extinction coefficient for H_2_O_2_ was *e* = 43.6/M/cm. Absolute enzyme activities were calculated by comparison with a standard curve generated by adding known amounts of purified catalase. Enzyme activity was then normalized to the protein content of each sample.

### 4.7. Superoxide Anion Radical (O_2_^−^) Scavenging Activity in a Suspension of Stimulated Cardiac Myocytes

An ESR spectrometer (JES-FR 30; JEOL, Tokyo, Japan) equipped with manganese oxide (MnO) as an internal standard was used for measuring O_2_^−^ scavenging activity. The conditions for ESR were as follows: magnetic field: 335 ± 5 mT; power: 4 mW; modulation width: 0.079 mT; modulation amplitude: 1 × 0.1 mT; response time: 0.1 s; amplitude: 1 × 200; sweep width: 5.000 mT; and sweep time: 2 min. The method for spin trapping of the O_2_^−^ radical was based on a previous study [18]. The reaction mixture consisted of 50 μL of 5 mM hypoxanthine (Sigma Chemical, St. Louis, MO, USA ), 15 μL of 5,5′-dimethyl-1-pyrroline-*N*-oxide (DMPO, Labotec Co., Tokyo, Japan), 50 μL of sample (*n* = 10 each) or superoxide dismutase (SOD)-bovine erythrocytes (Calbiochem, Inc., La Jolla, CA, USA), and 50 μL of 0.4 units/mL xanthine oxidase (XOD) (Roche K.K., Tokyo, Japan). After mixing well, the reaction mixture was transferred onto a quartz flat cell, and monitoring of the ESR spectrum was started exactly 1 min after the addition of XOD. All solutions were prepared with 0.1 M potassium phosphate buffer (pH 7.4). SOD activity in cardiac myocytes is shown as [(DMPO-O_2_^−^ signal before administration of sample – DMPO-O_2_^−^ signal after administration of sample/DMPO-O_2_^−^ signal before administration of sample) × 100%].

### 4.8. Measurement of Vessel Diameter

First, the control inner diameter of isolated coronary arterioles from WT mice was measured at 37 °C in a vessel chamber (2 mL) with an Olympus IX71 inverted microscope (vessel inner diameter: basal condition, 74 ± 8 μm; developed tone, 45 ± 6 μm). An aliquot of 100, 200, or 500 μL of supernatant (non-stimulated or stimulated) from WT or SMP30 KO cardiac myocytes was administered to suspend isolated coronary arterioles in a vessel bath. In our previous report, vasodilation occurred within 30 s after administration of the myocyte supernatant, reached a plateau within 1 min and was maintained for over 5 min. Thus, we measured the vessel inner diameter 5 min later, calculating any relative changes in inner diameter [10]. Each measurement was conducted using olmesartan (1 mM, Daiichi-Sankyo Co., Ltd., Tokyo, Japan) in the vessel bath and catalase (10,000 U) in the arterioles [18,19].

### 4.9. Data Analysis and Statistics

All statistical analyses were performed using StatView software (Abacus Concepts, Berkley, CA, USA). We used a one-way ANOVA followed by the Student-Neumann-Keuls *post hoc* test to compare echocardiographic data. We also used a one-way ANOVA followed by Tukey’s *post hoc* test to determine differences among the different interventions for H_2_O_2_, O_2_^−^ production and fluorescence intensity. A two-way ANOVA for repeated measures followed by Tukey’s *post hoc* test was used to determine differences in vasodilation resulting from various interventions. A paired *t*-test was used to determine the effects of catalase and olmesartan on arteriole diameter. Vascular diameters were normalized to the diameter with tone before the administration of the supernatant. Values are expressed as the mean ± S.E.M. Significance was set at *p* < 0.05 for all experiments.

## 5. Conclusions

Release of H_2_O_2_ and angiotensin via the redox pathway plays an important role in the SMP30 deficiency-related decline of metabolic coronary vasodilation caused by myocardium. These results provide useful information to clarify age- and oxidant-stress-related impairment of coronary flow regulation by myocardium.
